# Contextualizing Racial and Ethnic Inequities in COVID‐19 Vaccination Uptake During Pregnancy Through a Community‐Led Evaluation

**DOI:** 10.1002/bdr2.70093

**Published:** 2026-07-20

**Authors:** Hanna M. Shephard, Emma Posner, Sasha Rivera, Maudeline Auguste, Eirini Nestoridi, Melissa Colón, Michelle Thompson, Rebecca C. Fauth, Jessica L. Goldberg, Mahsa M. Yazdy

**Affiliations:** ^1^ Bureau of Family Health and Nutrition Massachusetts Department of Public Health Boston Massachusetts USA; ^2^ Department of Epidemiology University of Washington Seattle Washington USA; ^3^ Tufts Interdisciplinary Evaluation Research, Eliot‐Pearson Department of Child Study and Human Development Tufts University Medford Massachusetts USA

**Keywords:** community, COVID‐19, pregnancy, vaccination

## Abstract

**Background:**

Massachusetts Department of Public Health (MDPH) disaggregated data on vaccine coverage for pregnant people by their specific racial and ethnic identities, finding lower coverage among several subpopulations, including Puerto Rican and Haitian pregnant people. Quantitative data could not explain contexts shaping vaccine decisions; thus, MDPH partnered with Tufts Interdisciplinary Evaluation Research (TIER) to conduct a community‐led evaluation to understand COVID‐19 vaccine perceptions and decision‐making among Hispanic/Latina and Haitian residents pregnant when COVID‐19 vaccine access became widespread.

**Methods:**

The evaluation included a bilingual (English/Spanish) survey (*n* = 19) and in‐depth interviews (*n* = 8) conducted in Haitian Creole. TIER trained two community members who identified as Latina (Puerto Rican) and Haitian in evaluation methods and mentored them to collect, analyze, and interpret data, and co‐develop recommendations for improved community‐level vaccine outreach.

**Results:**

Two‐thirds of participants across subpopulations (18/27) reported receipt of at least one COVID‐19 vaccine dose. All but one Latina survey respondent received pregnancy‐specific vaccine information. While 10 received information from medical providers, only three indicated that providers helped them make COVID‐19 vaccine decisions. Most (*n* = 16) cited partners and family as helping them make vaccine decisions. In‐depth interviews with Haitian participants revealed concerns about the COVID‐19 vaccine including its safety in pregnancy, faith‐based objections, harm to Black communities, workplace mandates, and misinformation.

**Conclusions:**

Coupling disaggregated data with community‐led evaluation contextualized variations in vaccination during pregnancy. Strategies to support voluntary, informed vaccine decisions should emphasize provider communication, engage partners and families, and address concerns via trusted messengers. This replicable framework can support equitable outreach for vaccines recommended in pregnancy.

## 1 Background

Vaccination decision‐making, especially among pregnant people and those trying to conceive, is shaped by a complex interplay of personal, interpersonal, structural, and sociocultural factors. Before the COVID‐19 pandemic and vaccine rollout, research on the barriers and facilitators to vaccination during the perinatal period was limited. The pandemic revealed gaps in this understanding, as low uptake of COVID‐19 vaccines among pregnant people persisted (Galanis et al. 2022), despite elevated risk for severe disease from COVID‐19 among pregnant people and growing evidence of vaccine safety and effectiveness in reducing these risks (Chittajallu et al. 2025; Ciapponi et al. 2024; Fernández‐García et al. 2024; Ellington and Jatlaoui 2023). Subsequent studies identified several barriers to COVID‐19 vaccination during pregnancy, including lack of access to clear and culturally appropriate safety information, mistrust of medical and governmental institutions, fear of vaccine‐related adverse events, low perceived risk of infection, and political polarization surrounding the COVID‐19 pandemic (Casubhoy et al. 2024; Mitchell et al. 2023; Parsons et al. 2024). In contrast, key facilitators included the desire to protect oneself, one's baby, and the broader community, as well as receiving information from trusted sources (Casubhoy et al. 2024).

In the United States, stark racial and ethnic differences in COVID‐19 vaccination uptake during pregnancy have been documented; for example, non‐Hispanic Black pregnant individuals were 26% less likely to receive a COVID‐19 vaccine than non‐Hispanic White pregnant individuals (Razzaghi et al. 2022). Lower vaccine uptake among racially and ethnically minoritized pregnant people perpetuates inequities by concentrating severe disease in communities historically marginalized by healthcare systems (Khazanchi et al. 2020). Understanding not only who is less likely to be vaccinated, but also the underlying reasons for vaccine decision‐making is critical to designing equitable, effective public health interventions to promote vaccination during pregnancy.

To better characterize inequities, the Massachusetts Department of Public Health (MDPH) undertook a comprehensive descriptive analysis of COVID‐19 vaccination coverage during pregnancy, disaggregating vaccine rates using 12 racial and 34 ethnic identity categories. Detailed methods for MDPH's disaggregated analysis of COVID‐19 vaccination during pregnancy have been described previously (Shephard et al. 2023). Briefly, linked data from the Massachusetts Immunization Information System (Mass.gov, n.d.‐a) and birth certificates (Mass.gov, n.d.‐b) were analyzed to examine rates of COVID‐19 vaccination coverage before or during pregnancy, stratified by race and ethnicity. The race and ethnicity data, self‐reported on birth certificates, allowed individuals to select multiple categories and provide write‐in responses, enabling finer disaggregation than typical state‐ or national‐level datasets. MDPH's analysis reported that, as of October 2022, COVID‐19 vaccination coverage before or during pregnancy was 41.6% overall, but vaccination uptake was substantially lower among certain racial and ethnic subpopulations, including Puerto Rican (23.8%), Dominican (25.5%), Honduran (29.5%), and Haitian (26.7%) pregnant people, among other groups (Shephard et al. 2023; CDC Stacks, n.d.).

While this analysis revealed substantial variation in vaccination coverage among specific subgroups, quantitative data alone does not capture the nuanced experiences or sociocultural contexts influencing vaccine decision‐making during pregnancy. This information is crucial for informing effective interventions to promote equitable opportunities for vaccination among pregnant people. To address this gap, MDPH partnered with Tufts Interdisciplinary Evaluation Research (TIER) at Tufts University to conduct a community‐led participatory evaluation project aimed at shaping community‐informed interventions to reduce racial and ethnic inequities in COVID‐19 vaccination during pregnancy.

## 2 Methods

Using a participatory approach, TIER recruited residents with personal and/or professional experience connected to the project focus, trained these residents in evaluation, and mentored them to design and implement the project. Based on results from MDPH's disaggregated analysis showing lower rates of COVID‐19 vaccination coverage among certain ethnic groups, TIER distributed a recruitment call for community‐based evaluators through outreach to communities and networks connected to some of these groups. The two community‐based evaluators who were selected—S.R., who identifies as Puerto Rican, and M.A., who identifies as Haitian—were family support workers during the pandemic, are mothers, and are members of the communities their projects centered. The project aimed to understand where pregnant people receive information about the COVID‐19 vaccine, how they make decisions about the vaccine, their perspectives on the COVID‐19 vaccine, and how MDPH and other state and local public health initiatives can improve COVID‐19 vaccination efforts among pregnant people.

Each community‐based evaluator determined which method would be most appropriate for their focal population. One community‐based evaluator (S.R.) administered a survey to Latina mothers in Fall River to gather a broad range of perspectives from Fall River's diverse Latino community and to protect participant anonymity. The other community‐based evaluator (M.A.) conducted interviews with Haitian mothers in Brockton to facilitate access regardless of literacy and to build trust through face‐to‐face conversations.

### 2.1 Data Collection

#### 2.1.1 Survey of Latina Mothers

TIER programmed the 36‐item survey into Qualtrics in both English and Spanish. Recruitment materials were available in both languages and distributed via email, text, and in‐person to local organizations and networks such as family support programs and local Women, Infants and Children (WIC) offices. The survey included a built‐in set of screener questions to ensure that participants met eligibility criteria (i.e., pregnant at any time since May 2021 [when the vaccine became widely available in Massachusetts], identified as Latino/a/x, spoke English or Spanish, and lived in the greater Fall River area) before completing the survey. Participants received a $15 gift card for completing the survey, which was administered November 2022–March 2023 and completed by 19 people. Of the 19 survey respondents, 12 were born outside of the United States and 14 indicated Spanish as their preferred language. The largest proportion of respondents identified as Puerto Rican (6 of 19), with the remaining participants identifying as Brazilian, Dominican, Guatemalan, Ecuadorian, Honduran, Mexican, Latina (unspecified), and Salvadorean.

#### 2.1.2 Interviews With Haitian Mothers

M.A. conducted one‐on‐one interviews in Haitian Creole. She recruited eligible participants (i.e., pregnant at any time since May 2021 or currently pregnant, identified as Haitian, spoke Haitian Creole, and lived in the Brockton area) in‐person at a local family center, church, and stores frequented by the Haitian community. Participants received a $50 gift card for completing an interview. A total of eight interviews were completed between November 2022 and March 2023.

### 2.2 Data Analysis

With support from TIER mentors, the community‐based evaluators identified themes from their data, relative to the overall project aims and their own experiences. SR developed a survey analysis plan, and TIER cleaned and summarized the data descriptively in SPSS 28 using frequencies. Open‐ended survey responses were coded by the study team in Microsoft Excel. In collaboration with TIER mentors, S.R. reviewed analysis outputs for each evaluation aim to identify key patterns and emerging themes.

Interviews were audio recorded and transcribed first in Haitian Creole and then translated into English. M.A. and TIER mentors independently reviewed transcripts in Haitian Creole and English, respectively, and generated themes from each transcript and met to refine themes.

At the end of the analytic phase, the community‐based evaluators reviewed survey and interview findings together to generate community‐informed recommendations.

## 3 Results

Findings revealed relatively high uptake of the COVID‐19 vaccine among participants: two thirds (18 of 27) of survey (11 of 19) and interview (7 of 8) participants received at least one dose of the COVID‐19 vaccine. Despite high uptake, however, both survey and interview data uncovered underlying complexities and factors driving vaccine hesitancy among the two different populations.

### 3.1 Latina Mothers: Survey Findings

Among the Latina survey respondents, vaccination status varied by birthplace: 10 of the 12 respondents born outside of the United States or Puerto Rico received the COVID‐19 vaccine, compared to just 1 of the 7 respondents born in the United States or Puerto Rico.

**FIGURE 1:**
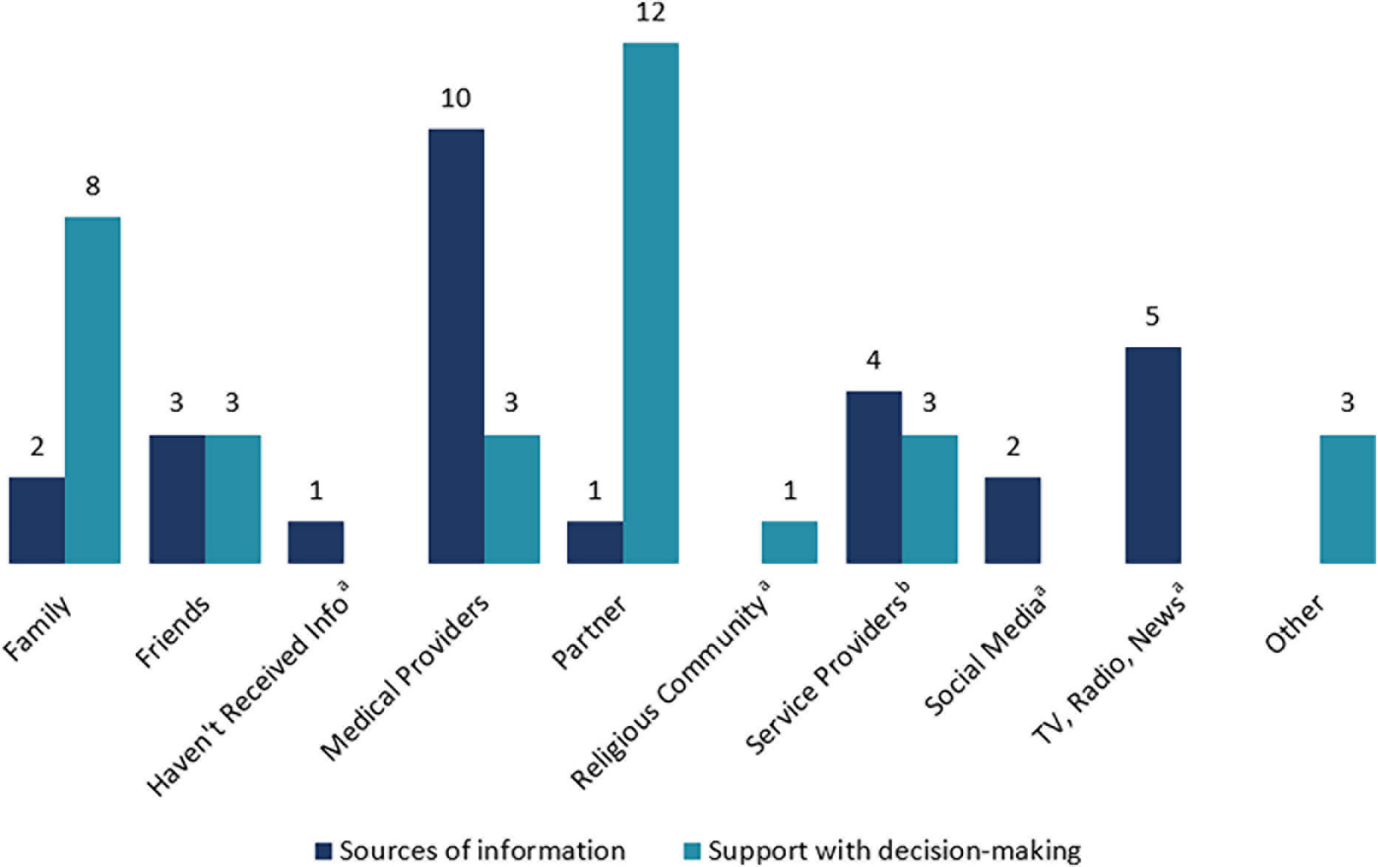
Source of information and decision‐making regarding the COVID‐19 vaccine based on survey of Latina mothers (*n* = 19). *Note:* For each question, participants could select more than one response. ^a^Some response options were not included in both questions, based upon context. ^b^Service providers include community organizations/programs, such as family support workers and programs, home visitors, social workers, parent mentors, and WIC.

All but one (18 out of 19) Latina survey respondents received information on COVID‐19 vaccination tailored for pregnant people. Latina mothers reported different sources of vaccine information compared to support for their decision‐making. Latina mothers identified medical providers as the most common source of information about the COVID‐19 vaccine, but just three respondents identified medical providers as supporting their decision‐making (see Figure 1). They identified partners (12 respondents) and family members (8 respondents) as the primary supports for their vaccine decision‐making, despite neither source being widely consulted as a source of information on vaccines (see Figure 1).

### 3.2 Haitian Mothers: Interview Findings

During interviews, many Haitian participants reported worrying about the short‐ and long‐term effects of the vaccine, even as many of these same women were aware of potential benefits. Participants described beliefs in their community that the vaccine is against their faith, and some people described more generalized fears that the vaccine is intended to harm Black people. Many participants shared how Haitian women believe their own natural remedies would protect them against, or help them fight, COVID‐19. They also shared other precautions—apart from the vaccine—that they felt more comfortable taking to minimize their risk of exposure to the virus, such as masking or distancing.

Many of the interviewees who were vaccinated shared that, despite concerns about the vaccine's safety, especially during pregnancy, they had no choice but to get vaccinated, some due to workplace mandates. Several interviewees spoke about widespread misinformation in their community and suggested women may be more trusting of information sources they already have a relationship with—for example, family members or church leaders.

### 3.3 Decision‐Making Factors Across Both Samples (Latina Mothers and Haitian Mothers)

Table 1 summarizes information provided by both groups on the factors affecting their vaccine decision‐making. As shown, there were strong commonalities across these distinct communities in those factors that promoted vaccination, such as workplace requirements and concern for their and others' health, and those that contributed to feelings of hesitancy, including apprehensions about vaccine safety, lack of clear information on side effects and long‐term impacts, and myths and misconceptions about the vaccine.

**TABLE 1:** Factors affecting COVID‐19 vaccine decision‐making.

Factors affecting vaccine decision‐making		Latina mothers	Haitian mothers
Promoting factors	Workplace requirements	✓	✓
	Vaccine protection against COVID‐19 for self/baby	✓	✓
	Protecting others from COVID‐19	✓	✓
Factors contributing to hesitancy	Fears or concerns about vaccine safety or side effects	✓	✓
	Lack of information on long‐term effects	✓	✓
	Vaccine myths or misconceptions	✓	✓
	Religious beliefs	—	✓
	Workplace requirements	✓	✓
	Preference for other precautions, such as masking, social distancing, natural remedies, or antibodies from recent COVID‐19 infection	✓	✓

*Note:* Findings from the Latina mothers are based on open‐ended survey items coded by the study team.

### 3.4 Recommendations

Based on the findings from this project, community‐based evaluators developed the following recommendations for state and local public health initiatives to improve vaccination coverage during pregnancy:

- Build capacity among medical providers, family support workers, and trusted community leaders who interface with pregnant people to provide comprehensive information about benefits and disadvantages of the vaccine, side effects, and effects on pregnant people and children in language that is easily understood.
- Create and facilitate opportunities for pregnant people to ask questions to medical providers, community leaders, and community members who have received the vaccine, and engage their broader support systems, including family members and partners, to help navigate vaccine decision‐making.
- Focus vaccine outreach efforts on trusted locations, such as churches, early childhood and family support programs, healthcare settings, public schools, or places where communities gather, and recognize that these locations may vary by community, group, and individual.

## 4 Discussion

COVID‐19 vaccination decision‐making during pregnancy is complex, requiring solutions informed by the people most impacted by public health challenges. This project illustrates how combining disaggregated quantitative data with community‐led evaluation can generate nuanced insights into inequities in vaccine coverage.

Through this participatory evaluation approach, community members led data collection and interpretation, ensuring recommendations were grounded in lived experiences and cultural contexts. This approach not only reveals the multifaceted barriers and facilitators surrounding vaccination but also builds trust and capacity within communities historically marginalized by healthcare systems. Our findings reinforce the critical role of trusted interpersonal relationships and culturally responsive communication in vaccine acceptance during pregnancy. Pregnant people do not make decisions in isolation; partners, family, employers, and community narratives shape perceptions of risk and benefit. The sense of pressure or lack of choice reported by some participants risks exacerbating public health mistrust and underscores the importance of voluntary, informed decision‐making supported by respectful dialogue.

As additional vaccines are recommended for pregnant populations (e.g., respiratory syncytial virus [RSV], influenza), public health systems must invest in community‐informed strategies that prioritize transparency, address concerns, and leverage trusted local partners. This need is especially acute given the rise of vaccine misinformation amplified on social media targeting pregnant people during the COVID‐19 pandemic (Malik et al. 2024; Jheng et al. 2025). The disaggregated data analysis followed by community‐led evaluation offers a replicable framework for such strategies, bridging gaps between epidemiologic data and lived realities.

Limitations included the small survey sample size and the use of distinct data collection methods in just two communities. The survey response was low in part due to COVID‐19‐related survey fatigue and initial reliance on virtual outreach through third parties, such as community organizations. Pivoting to in‐person outreach in community settings, as used for interview recruitment, yielded higher participation. Future survey administration should prioritize in‐person outreach and offer alternate modes of completion beyond online self‐report methods, which rely on internet or online access and literacy skills to read and understand the survey items. Options such as in‐person, read‐aloud survey administration could address potential literacy and technology barriers. Further, although each method offered distinct capacity‐building opportunities for the community‐based evaluation team, the use of both a survey and interviews limited the potential for a more cohesive analysis. Qualitative data gleaned from interviews were particularly well‐positioned to inform community‐specific recommendations on vaccine outreach and may be best suited for this topic. Finally, this project was conducted in just two communities. There remains a need for further research across additional racial and ethnic subgroups, including Indigenous populations.

Ultimately, strategies to increase vaccine confidence during pregnancy require strengthening healthcare provider communication, leveraging trusted messengers to deliver accessible and tailored information, and cultivating culturally and linguistically inclusive spaces where pregnant people and their inner networks feel heard, respected, and supported to make decisions. Holistic, community‐centered approaches are vital to addressing racial and ethnic inequities in vaccination during the perinatal period, a critical window marked by heightened vaccine hesitancy and vulnerability.

## Author Contributions

**Hanna M. Shephard:** writing, editing, conceptualization. **Emma Posner** and **Rebecca C. Fauth:** writing, reviewing, editing, conceptualization, data analysis, community evaluation mentoring. **Jessica L. Goldberg** and **Melissa Colón:** reviewing, editing, conceptualization, community evaluation mentoring. **Michelle Thompson:** data analysis, community evaluation mentoring. **Eirini Nestoridi** and **Mahsa M. Yazdy:** reviewing, editing, conceptualization. **Sasha Rivera** and **Maudeline Auguste:** study design, data collection, data analysis, interpretation of data analysis.

## Funding

This study was supported by funds made available from the Centers for Disease Control and Prevention, Center for State, Tribal, Local and Territorial Support, under grant #6 NH75OT000030‐01‐03.

## Conflicts of Interest

The authors declare no conflicts of interest.

## Data Availability

The data that support the findings of this study are available from the corresponding author upon reasonable request.

## Appendix A Survey

### Eligibility Screener

**The next set of questions asks whether you meet the eligibility criteria to take this survey**.

1. How old are you?

________________________________________________________________

2 Where do you currently live?
  ○ Fall River
  ○ Swansea
  ○ Somerset
  ○ Westport
  ○ Other city or town (please specify) __________________________________________________
3 Are you currently pregnant?
  ○ Yes
  ○ No
  ○ I don't know
3.1 *[IF ANSWER TO QUESTION 3 WAS “No” or “I don't know”]* Were you pregnant at any point between May 2021 and now? For example, if your youngest child is *less than* 22 months old.
  ○ Yes
  ○ No
4 Are you Latina/Latino or Hispanic?
  ○ Yes
  ○ No
  *[IF ANSWER TO QUESTION 1 WAS LESS THAN 18 OR IF ANSWER TO QUESTION 3.1 OR 4 WAS “No”]* Thank you for your interest in our survey! Based on your responses, we've determined that you do not meet the eligibility criteria for this study. We appreciate your time! *[SKIP TO END OF SURVEY]*

### Demographics

**The next set of questions asks a little bit about you and your family**.

5 What is your ethnic and/or national background? (Some examples include but are not limited to: Dominican, Ecuadorian, Guatemalan, Puerto Rican).
  __________________________________________________
6 Which categories describe you? (Select all that apply)
  □ Asian
  □ Black, African, or African American
  □ Native American or Alaska Native
  □ Native Hawaiian or Pacific Islander
  □ White
  □ Other (please specify all) __________________________________________________.
  □ Prefer not to answer
7 How many years have you lived in the United States?
  ○ Less than a year
  ○ 1–5 years
  ○ More than 5 years
8 Were you born in the United States?
  ○ Yes
  ○ No
9 Were you born in Puerto Rico?
  ○ Yes
  ○ No
10 Please tell us the year you were born.
  _________________________________________________
11 Please describe your gender.
  ○ Man
  ○ Woman
  ○ Non‐Binary
  ○ Transgender Man
  ○ Transgender Woman
  ○ Other (please specify) __________________________________________________
  ○ Prefer not to answer
12 What is/are your preferred language(s)? (Select all that apply)
  □ Spanish
  □ English
  □ Portuguese
  □ Other (please specify) __________________________________________________
13 What is your highest level of education?
  ○ Less than high school
  ○ GED/HiSET
  ○ High School Graduate
  ○ Some College
  ○ Associate's Degree or Technical Degree (i.e., Cosmetology, electrician)
  ○ Bachelor's Degree
  ○ Master's Degree or higher
  ○ Other (please specify) __________________________________________________
14 What is your marital status?
  ○ Single
  ○ Not Married, but in a Relationship
  ○ Married
  ○ Separated or Divorced
  ○ Widowed
  ○ Other (please specify) __________________________________________________
15 How many children (under the age of 18) are currently in your care?
  __________________________________________________
16 Think about your [current OR most recent] pregnancy. [Is OR Was] this your first pregnancy?
  ○ Yes
  ○ No
  ○ I don't know
17 Did you have a primary care provider BEFORE your pregnancy?
  ○ Yes
  ○ No

### Pregnancy

*[IF RECENTLY PREGNANT]* **The following questions are about your experiences while pregnant. If you have had more than one pregnancy since May 2021, answer these questions about your most recent pregnancy**.

*[IF CURRENTLY PREGNANT]* **The following questions are about your experiences during your current pregnancy**.

18 *[IF* *RECENTLY* *PREGNANT]* What was the delivery date of your most recent pregnancy? *[IF* *CURRENTLY* *PREGNANT]* What is your current due date?
  Month: *[DROPDOWN MENU OF MONTHS]*
  Day: *[DROPDOWN MENU OF DAYS]*
  Year: *[DROPDOWN MENU OF YEARS]*
19 From whom [did you receive OR have you been receiving] your prenatal care? (Select all that apply)
  □ Emergency Room Provider
  □ Primary Care Provider
  □ OB‐GYN
  □ Doula
  □ Midwife
  □ I did not receive prenatal care
  □ Other (please specify) __________________________________________________
19.1 *[IF ANSWER TO QUESTION 19 WAS NOT* “*I did not receive prenatal care*”*]* Please tell us the name of the practice where you [received OR have been receiving] prenatal care. For example, Truesdale OB‐GYN, Highland OB‐GYN, or another practice.
19.2 *[IF ANSWER TO QUESTION 19 WAS NOT* “*I did not receive prenatal care*”*]* During what trimester of your pregnancy did you begin prenatal care?
  ○ First trimester (0–3 months)
  ○ Second trimester (4–6 months)
  ○ Third trimester (7–9+ months)
19.3 *[IF* *ANSWER TO* *QUESTION 19 WAS NOT “I did not receive prenatal care”]* [Did you receive OR Have you been receiving] your prenatal care in a language you feel comfortable with?
  ○ Yes, I received ***all*** of my prenatal care in a language I'm comfortable with.
  ○ Yes, I received ***some*** of my prenatal care in a language I'm comfortable with.
  ○ No, I received ***none*** of my prenatal care in a language I'm comfortable with.
19.4 *[IF* *ANSWER TO* *QUESTION 19 WAS NOT “I did not receive prenatal care”]* In what language(s) [did you receive OR have you been receiving] your prenatal care? (Select all that apply)
  □ English
  □ Spanish
  □ Portuguese
  □ Other (please specify) __________________________________________________
20 Did you have any pre‐existing health conditions before your [most recent OR current] pregnancy? For example, hypertension (high blood pressure), overweight, underweight, asthma, other bronchial conditions, or other pre‐existing health conditions.
  ○ Yes
  ○ No
21 During your [most recent OR current] pregnancy, who helped you make decisions regarding your pregnancy and baby? (Select all that apply)
  □ Partner
  □ Family members
  □ Friends
  □ Medical providers (for example, prenatal care provider, doula, visiting nurse, primary care provider, emergency room provider)
  □ Service providers and community organization or programs (for example, family support workers and programs, home visitors, social workers, parent mentors, WIC)
  □ My religious community (e.g., pastor)
  □ People in my Support Groups (for example, Mommy and Me or First Time Mom groups)
  □ Other (please specify) __________________________________________________

### Vaccinations

**In this section, we want to know more about your experiences related to common vaccinations**.

22 As far as you know, were you up to date with the regularly recommended vaccinations before becoming pregnant? For example, tetanus, diphtheria, pertussis (Tdap) vaccine, hepatitis B vaccine, etc.
  ○ Yes
  ○ No
  ○ I don't know
23 [Have you received OR Did you receive] a flu shot during your [current OR most recent] pregnancy?
  ○ Yes
  ○ No
  ○ I don't know
24 *[IF* *CURRENTLY PREGNANT AND ANSWER TO* *QUESTION 23 WAS “No”]* Do you plan to receive a flu shot while you are pregnant?
  ○ Yes
  ○ No
  ○ I'm not sure
  **These next questions ask about your experiences with the COVID‐19 vaccine**.
25 Where did you get information regarding the COVID‐19 vaccination for people who are pregnant? (Select all that apply)
  □ Partner
  □ Family members
  □ Friends
  □ Medical providers (for example, prenatal care provider, doula, visiting nurse, primary care provider, emergency room provider)
  □ Service providers and community organization or programs (for example, family support workers and programs, home visitors, social workers, parent mentors, WIC)
  □ My religious community (e.g., pastor)
  □ People in my support groups (for example, Mommy and Me or First Time Mom groups)
  □ Community resource fair
  □ Social media or online forums
  □ Television, radio and newspapers articles
  □ Other (please specify) __________________________________________________
  □ I have not received information on the COVID‐19 vaccine for people who are pregnant
26 *[IF* *ANSWER TO* *QUESTION 25 WAS NOT “I have not received information on the COVID‐19 vaccine for people who are pregnant*”*]* To your knowledge, did any of the information you received come from the following sources? (Select all that apply)
  □ Centers for Disease Control and Prevention (CDC)
  □ Massachusetts Department of Public Health (MDPH)
  □ Local board of health or my city/town's health department
  □ I don't know
27 *[IF* *ANSWER TO* *QUESTION 25 WAS NOT “I have not received information on the COVID‐19 vaccine for people who are pregnant”]* In which language(s) did you receive information regarding the COVID‐19 vaccination for people who are pregnant? (Select all that apply)
  □ English
  □ Spanish
  □ Portuguese
  □ Other (please specify) __________________________________________________
28 *[IF* *ANSWER TO* *QUESTION 19 WAS NOT “I did not receive prenatal care”]* [Did OR Has] your prenatal care provider discuss[ed] the COVID‐19 vaccine with you?
  ○ Yes
  ○ No
29 *[IF ANSWER TO QUESTION 28 WAS “Yes”]* Please rate your comfort level when discussing the COVID‐19 vaccination with your prenatal provider
  ○ Very comfortable
  ○ Comfortable
  ○ Neither comfortable nor uncomfortable
  ○ Uncomfortable
  ○ Very uncomfortable
30 *[IF ANSWER TO QUESTION 28 WAS “No”]* How comfortable would you [be OR have been] discussing the COVID‐19 vaccine with your prenatal care provider?
  ○ Very comfortable
  ○ Comfortable
  ○ Neither comfortable nor uncomfortable
  ○ Uncomfortable
  ○ Very uncomfortable
31 *[IF ANSWER TO QUESTION 28 WAS “Yes”]* Think about your [current OR most recent] pregnancy. [Do OR Did] you feel your prenatal provider addressed your questions or concerns about the COVID‐19 vaccination?
  ○ All of my questions or concerns were addressed.
  ○ Some of my questions or concerns were addressed.
  ○ None of my questions or concerns were addressed.
  ○ I didn't have any questions or concerns.
32 Have any of the following helped you make decisions about the COVID‐19 vaccine? (Select all that apply)
  □ Partner
  □ Family members
  □ Friends
  □ Medical providers (for example, prenatal care provider, doula, visiting nurse, primary care provider, emergency room provider)
  □ Service providers and community organization or programs (for example, family support workers and programs, home visitors, social workers, parent mentors, WIC)
  □ My religious community (e.g., pastor)
  □ People in my support groups (for example, Mommy and Me or First Time Mom groups)
  □ Community resource fair
  □ Social media or online forums
  □ Television, radio, and newspapers articles
  □ Other (please specify) __________________________________________________
  □ None—No one helped me make decisions about the vaccine
33 Have you received *any* COVID‐19 vaccination?
  ○ Yes
  ○ No
33.1 *[IF* *CURRENTLY PREGNANT AND ANSWER TO* *QUESTION 33 WAS “Yes”]* When did you receive your first COVID‐19 vaccination dose?
  ○ Before my current pregnancy
  ○ During my ***current*** pregnancy
  ○ During a ***previous*** pregnancy
  ○ Other (please specify) __________________________________________________
33.2 *[IF* *RECENTLY PREGNANT AND ANSWER TO* *QUESTION 33 WAS “Yes”]* When did you receive your first COVID‐19 vaccination dose?
  ○ Before my Pregnancy
  ○ During my Pregnancy
  ○ After Giving Birth
  ○ Other (please specify) __________________________________________________
33.3 *[IF* *ANSWER TO* *QUESTION 33 WAS “No”]* Do you plan to get vaccinated against COVID‐19?
  ○ Yes, during my pregnancy
  ○ Yes, after giving birth
  ○ No
  ○ I haven't decided
34 *[IF* *VACCINATED OR PLANNING TO BE* *VACCINATED]* In a sentence or two, please explain why you decided to get vaccinated against COVID‐19 [before pregnancy OR during pregnancy OR after giving birth].
  _________________________________________________

### For Unvaccinated

35 *[IF ANSWER TO QUESTION 33.3 WAS “I haven't decided”]* In a sentence or two, please tell us why you haven't decided whether to get vaccinated against COVID‐19.
36 *[IF ANSWERS TO QUESTIONS 33 & 33.3 WERE “No”]* In a sentence or two, please tell us why you decided not to get vaccinated against COVID‐19.

### For Vaccinated

37 *[IF* *ANSWER TO* *QUESTION 33 WAS “Yes”]* How would you describe your COVID‐19 vaccination status?
  ○ I am partially vaccinated (for example, I have received one dose of Pfizer or Moderna)
  ○ I am fully vaccinated but not boosted (for example, I have received two doses of Pfizer or Moderna, or one dose of Johnson & Johnson)
  ○ I am fully vaccinated and have received at least one booster shot
  ○ I don't know
38 *[IF* *ANSWER TO* *QUESTION 33 WAS “Yes”]* What season and year was your first COVID‐19 vaccination dose?
  *[DROPDOWN MENU WITH SEASONS/YEARS]*
39 *[IF* *ANSWER TO* *QUESTION 33.1 WAS “During my current pregnancy” OR “During a previous pregnancy” OR* *IF ANSWER TO* *QUESTION 33.2 WAS “During my Pregnancy”]* During what trimester did you get the first dose of the COVID‐19 vaccine?
  ○ First trimester (0–3 months)
  ○ Second trimester (4–6 months)
  ○ Third trimester (7–9+ months)
  ○ I don't remember

### Closing

40 Did anyone in your close circle (for example, family members, friends) receive a COVID‐19 vaccination during their pregnancy?
  ○ Yes
  ○ No
  ○ I don't know
  **These last two questions ask for your perspective on how the Massachusetts Department of Public Health (MDPH) can address the barriers or concerns regarding the COVID‐19 vaccination among pregnant Latina/o or Hispanic people. The Centers for Disease Control and Prevention (CDC) and health care providers recommend that all pregnant people receive the COVID‐19 vaccination. Yet, Latina/o or Hispanic people who are pregnant have some of the lowest vaccination rates**.
41 In a sentence or two, tell us why you think pregnant Latina/o or Hispanic people have low rates of vaccination against COVID‐19.
  _________________________________________________
42 In a sentence or two, tell us what you think can be done to increase comfort with the COVID‐19 vaccine among pregnant people in the Latina/o or Hispanic community.
  _________________________________________________

## Appendix B Interview Guide

### Pandemic

**We want to understand the experiences of Haitian Women who were pregnant during the pandemic and their thoughts on the vaccine. Let's start by talking about the pandemic**.

1. Where were you living in March 2020? Were you working or in school at that time?
  1a. How did you first find out about the pandemic?
2. How did you initially view the COVID‐19 virus? How do you view it now?

### Pregnancy

**Next, we'll discuss your pregnancy during the COVID‐19 pandemic**.

3 Tell me about when and where your baby was born. Was this your first baby?
4 What was it like to be pregnant during the pandemic? What concerns did you have for yourself or your baby?
5 Where did you get prenatal care? How did you choose this place?
  5a How did you feel when you went to [that hospital/clinic] during that time?
  5b Did they have any special rules or restrictions because of COVID‐19?
6 How did it feel to have an infant during the pandemic?
7 Overall, how do you feel about the care you received while you were pregnant?

### COVID‐19 Vaccine

**Now, let's talk about the COVID‐19 vaccine. The vaccine became widely available for pregnant people in May 2021**.

8 How did you find out that pregnant people could be vaccinated against COVID‐19? How did you feel about it?
9 Do you know anyone who received a vaccine while pregnant?
10 Did you receive the vaccine while pregnant? Why or why not?
  10a How did you make your decision? How did you feel about your decision?
  10b [If they did NOT get vaccinated while pregnant]: Did you receive the vaccine after you were pregnant? Why or why not?
11 What did your family or friends think about your decision to get vaccinated or not get vaccinated?

### Closing Questions/Recommendations

12 Haitian women have some of the lowest vaccination rates in Massachusetts. Why do you think that Haitian women have low rates of vaccination against COVID‐19?
13 What do you think can be done to increase Haitian women's comfort with the vaccine?
